# Effects of Dietary Fatty Acids in Pancreatic Beta Cell Metabolism, Implications in Homeostasis

**DOI:** 10.3390/nu10040393

**Published:** 2018-03-22

**Authors:** Paloma Acosta-Montaño, Víctor García-González

**Affiliations:** Departamento de Bioquímica, Facultad de Medicina Mexicali, Universidad Autónoma de Baja California, Mexicali 21000, Mexico; paloma.acosta@uabc.edu.mx

**Keywords:** fatty acids, type 2 diabetes, lipotoxicity, homeostasis

## Abstract

Fatty acids are involved in several metabolic processes, including the development of metabolic and cardiovascular diseases. In recent years a disease that has received escalated interest is type 2 diabetes (T2D). Many contributing factors including a high-caloric diet rich in dietary saturated fats have been broadly characterized as triggers of T2D. Insulin resistance resulting from a high saturated fat diet leads to alterations in lipid cellular intake and accumulation which generate lipotoxic conditions, a key phenomenon in the metabolism of β-cells. Alternatively, unsaturated fatty acids have been described to show opposite effects in pancreatic β-cells. The purpose of this work is to perform a critical analysis of the complex role of saturated and unsaturated fatty acids in β-cell metabolism. We discuss the diverse effects main dietary fatty acids have upon pancreatic β-cell metabolism as a key factor to maintain homeostasis by focusing in the cellular and molecular mechanisms involved in the development and progression of T2D. For instance, modifications in protein homeostasis as well as the intracellular management of lipid metabolism which are associated with inflammatory pathways. These conditions initiate critical metabolic rearrangements, that in turn have repercussions on insulin β-cell metabolism. This review allows an integral and broad understanding of different functions of fatty acids inside β-cells, being important metabolites for novel therapeutic targets in T2D treatment.

## 1. Introduction

Fatty acids (FAs) are essential components of the human diet and are obtained from two main sources: animals and plants. The difference lies in the FA composition. Animal fats are rich in saturated fatty acids (SFAs), whereas lipids obtained from plants contain unsaturated fatty acids (UFAs), that can be classified as mono (MUFAs) or polyunsaturated (PUFAs) [1]. Several studies associate dietary FAs with prevention and progression of non-transmissible chronic diseases such as type 2 diabetes (T2D) and cardiovascular diseases [2]. The study of these pathologies is relevant because said pathologies form part of the main causes of death. According to the World Health Organization, in 2015 the main causes of death were ischemic heart disease and stroke, which together reported 15 million deaths around the world. These diseases have remained the leading causes of death globally since 2000. T2D was listed as the sixth main cause of death accounting for 1.58 million lives [3]. The International Diabetes Federation calculated approximately 4 million deaths from diabetes in 2017 and estimated 425 million adults with diabetes worldwide. This means that 1 out of every 11 adults has diabetes [4], of which the most common type is T2D accounting for around 90–95% of all cases [5]. Furthermore, an additional 352 million adults have impaired glucose tolerance. Added to the original 425 million adults diagnosed with diabetes, this has a cost of nearly 727 billion dollars worldwide in patients between 20 and 79 years old. For instance, Mexico is the second Latin American country and fifth in the world in prevalence of diabetes with nearly 12 million patients [4]. Due to all of this, diabetes has become a major public health problem internationally.

T2D is the main type of diabetes and its complex etiology combines environmental and genetic factors. This disease is a result of a high demand of insulin synthesis in pancreatic β-cells caused by hypercaloric diets and lifestyle conditions such as the lack of physical activity which produces insulin resistance in the liver and insulin-dependent tissues including adipose tissue and muscle. Pancreatic β-cells are capable of sensing glucose fluctuations and in response release insulin, a hormone that is responsible for stimulating glucose uptake in the muscle and adipose tissue normalizing plasmatic glucose [6]. It is estimated that upon receiving a glucose stimulus, the endoplasmic reticulum (ER) of each β-cell synthetizes close to one million insulin molecules per minute packed in small granules. Each β-cell contains approximately 10,000 granules, of which only 0.14%/min (first phase) and 0.05%/min (second phase) is released upon stimuli [7,8,9]. The phenomenon of glucose-stimulated insulin secretion (GSIS) is biphasic. The first phase results from the triggering of ATP-sensitive K^+^ channel-dependent that increases cytoplasmic Ca^2+^ and discharges from a readily releasable pool of granules that are in contact with the plasma membrane [10]. The release rate of this first phase is around 15 granules per minute lasting between 5 and 10 min for each β-cell [7]. The second phase entails the preparation, translocation and priming of granules (reserve pool) for release, also triggered by elevated intracellular Ca^2+^ levels given by the K^+^(ATP) channel-dependent pathway [10]. In this second phase, the release rate decreases to five granules per minute [7].

An increase in protein load for insulin synthesis in β-cells due to hyperglycemic conditions can result in β-cell expansion and generate hyperinsulinemia as a compensatory mechanism. However, this process gradually leads to β-cell mass loss, generating ER stress [11]. Conditions that disrupt metabolic homeostasis cause distension of ER cisterns, affecting protein folding [12] and alteration of post-translational modifications. As a result, the ER generates an adaptive response known as the unfolded protein response (UPR). This pathway consists of three major signaling transducers initiated by protein kinase RNA-like endoplasmic reticulum kinase (PERK), activating transcription factor 6 (ATF6) and a serine/threonine-protein kinase/endoribonuclease the inositol-requiring enzyme 1 (IRE1), which function as sensors of alterations in the load of misfolded proteins in the ER lumen [13]. This pathway as a whole is responsible for enhancing folding capacity by increasing the production of chaperones and enzymes of protein maturation [8]. This phenomenon is followed by the reduction of the ER overload by decreasing mRNA translation and improving mRNA degradation by an increase of ER-associated degradation proteins and components of autophagy to promote elimination of unfolded and aggregated proteins [14]. The UPR pathway is critical, since it is part of the cellular response to modulate general cell homeostasis [15] by regulating insulin synthesis and consequently plasmatic glucose as well as the energetic metabolism.

Importantly, chronic exposure to high levels of free fatty acids (FFAs) leads to lipotoxicity [16]. In high-fat diets, adipose tissue storage capacity for triacyclglycerols (TAGs) can be overloaded. Lipotoxicity describes the deleterious effects that lipid accumulation can cause in peripheral tissues. This condition has been recognized as a contributing factor to the development of T2D, characterized by the loss of β-cells functionality that eventually leads to cellular apoptosis. This phenomenon is described as lipoapoptosis [17].

Evidence suggests FFAs play a specific role in β-cells, however, some mechanisms by which FFAs exert their harmful or even beneficial effects remain to be elucidated. Furthermore, the properties of each FFA provide particular functions in β-cells, such as chain length, number or position of double bonds, affinity and interaction with other FFAs as well as with the cell itself. In this review, we describe the impact that FFAs have on β-cells homeostasis focusing on their metabolism and molecular effects.

## 2. Fatty Acids Properties

SFAs have been associated with adverse health effects, including palmitic acid (16:0, PA), myristic acid (14:0, MA) and stearic acid (18:0, SA). Palmitic acid is the most common saturated fatty acid found in the human body, representing 20–30% of total FAs in membrane phospholipids and adipose tissue TAGs, and on average a 70 kg man is made up of 3.5 kg of PA [18], with an intake of approximately 20–30 g/day [19]. Palmitate can be obtained in the diet or synthesized endogenously from fatty acids, carbohydrates and amino acid metabolism. It is a major component of palm oil (44% of total fats), and can also be found in meat and dairy products (50–60% of total fats), as well as cocoa butter (26%) and olive oil (8–20%) [20].

**Figure 1:**
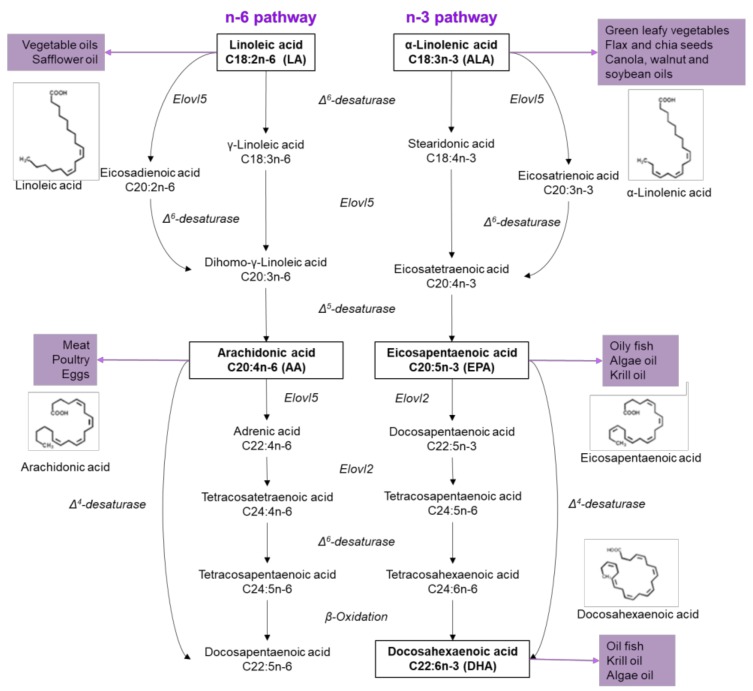
Sources and metabolism of n-6 and n-3 fatty acids. On the left side, the pathway of n-6 FA is described, which begins with linoleic acid to generate arachidonic acid through the enzyme Δ^5^-desaturase. On the right side, the n-3 FA pathway starts with linolenic acid and eicosapentaenoic acid is obtained through two steps, competing for the Δ^5^-desaturase enzyme in n-6 pathway. Subsequently, docosahexaenoic acid is generated by four consequent reactions.

On the other hand, UFAs are generally related to protective effects, like preventing β-cell apoptosis, regulating plasmatic glucose concentrations and enhancing insulin sensitivity, and can be classified into MUFAs and PUFAs. The first ones include oleic (18:1, OA) and palmitoleic acid (16:1, PAO), and can be found in several animal and vegetable oils. Also, it can be obtained from the regulation of palmitate accumulation, which under normal physiological conditions is prevented by enhanced Δ^9^ desaturation to PAO and/or elongation to stearic acid, and further Δ^9^ desaturation to OA [20]. PUFAs are hydrocarbon chains with two or more double bonds located along the chain (Figure 1) [2]. Depending in the location of the first double bond and according to the methyl group, UFAs are classified as n-6 or n-3. α-Linolenic acid (ALA; 18: 3n-3) initiates n-3 PUFA, an essential FA found in leafy vegetables, nuts, soybeans, flaxseed, chia and vegetable oils. Linoleic acid (LA; 18: 2n-6) is the precursor of n-6 PUFA, also an essential FA that cannot be synthesized by mammals, found in vegetable oils, seeds and nuts. Both fatty acids, linoleic and linolenic, are metabolized through desaturation and elongation reactions. LA is metabolized into arachidonic acid (AA; 20: 4n-6); while ALA in eicosapentaenoic acid (EPA; 20: 5n-3) and, finally, in docosahexaenoic acid (DHA; 22: 6n-3) (Figure 1) [2]. Importantly, EPA and DHA are obtained from fish and fish oil supplements, as well as other marine products [21], as a result of plankton and algae consumption [22].

## 3. Fatty Acid Metabolism in β-Cells

Humans can synthesize SFAs and de novo MUFAs, however, we lack the enzymes to incorporate a double bond at position n-3 or n-6 of the fatty acid [22]; consequently, they are considered essentials. ALA and LA have a common metabolic pathway, therefore, competing for enzymes such as Elovl5, an elongase, and for the Δ^6^-desaturase (D6D) (Figure 1) [23]. However, D6D activity is higher in n-3 than in n-6 PUFAS, suggesting n-3 PUFAs are more rapidly synthesized than n-6 PUFAs, since both substrates (ALA and LA) compete for active sites on D6D. ALA is also the preferred substrate due to an affinity two to three times higher for ALA than of LA [24,25]. Ingested PUFAS could have several metabolic fates, including β-oxidation, carbon recycling and direct incorporation into structural lipids [26].

Several studies show that β-oxidation normally consumes the majority of linoleate and α-linolenate intake, accounting for 65–85% of their intake, rising up to 100% during energy deficit; besides, PUFAs are more easily β-oxidized than saturated, as seen in hepatocytes and neonatal metabolism [27,28]. Also, LA and ALA normally accumulate in skin, muscle and adipose tissue, but LA tissue concentration is higher in the liver [29]. However, significant components of the carbon backbone from LA and ALA that are not completely β-oxidized are incorporated into newly synthesized cholesterol or fatty acids through acetyl CoA or acetoacetate [29,30]. Nonetheless, studies are needed to elucidate n-6 PUFA metabolism in β-cells.

**Figure 2:**
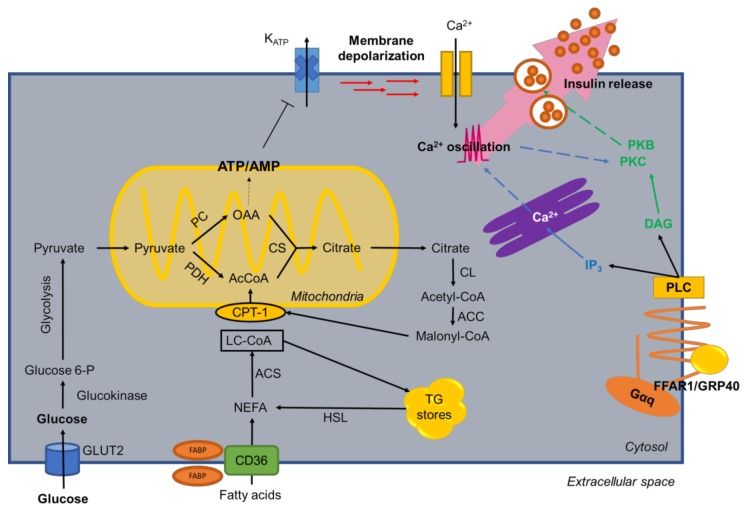
General metabolism of fatty acids in β-cells. The main metabolic pathways of FFAs are outlined in the figure, such as glycolysis, FA biosynthesis, β-oxidation. Glycolysis produces an increase in ATP, which closes K(ATP)-dependent channels and causes membrane depolarization and the opening of voltage-dependent Ca^2+^ channels, stimulating insulin release. Also, the binding of fatty acids to free fatty acid receptor 1 (FFAR1) generates changes of Ca^2+^ in ER lumen promoting insulin release in β-cell.

Under fasting conditions, fatty acids are the main source of endogenous energy in β-cells [31]. These cells have a low glycogen reserve; almost all glucose entering the β-cell is oxidized via glycolysis and mitochondrial oxidative phosphorylation to ATP, only <10% of glucose uptake accounts for glycogen synthesis [32]. Also, these cells maintain high levels of oxygen consumption in the absence of glucose [33]. In a state where glycemia is below basal levels, fatty acids are converted by acyl-CoA synthetase (ACS) into long-chain acyl-CoA and enter the mitochondria for β-oxidation for energy production [33]. An increase in glycemia, usually after a meal, decreases fatty acid oxidation, increasing the catabolism of glucose [34]. Glucose metabolism causes an increase in intracellular levels of long chain acyl-CoA [35], augmentation of the conversion of glucose to malonyl-CoA, promotion of nutrient storage [36] and an inhibition of CPT-1, hence, blocking the oxidation of fatty acids [33]. Low levels of de novo fatty acid synthesis within the β-cell indicates malonyl-CoA is been used as a “switch” metabolite, instead of a precursor (Figure 2) [33].

Internalization of FAs into cells is vital for cellular metabolism, including their incorporation into the phospholipids of plasmatic and specific organelle membranes [37]. Glucose and amino acids are known to cross the plasma membrane via transmembrane transport proteins [38]. Exogenous FAs enter the cell primarily by facilitated transport [39] mediated by plasma membrane fatty acid-binding proteins (FABPs) (Figure 2), which facilitate the dissociation of fatty acids from albumin [40]. A fatty acid transporter, CD36, with high affinity for long chain fatty acids [41] has been identified in the plasma membrane of muscle, liver and platelet cells [42,43]; indeed, expression has been demonstrated in human pancreatic cells [39]. Upon fatty acid entry, cytoplasmic FABPs bind one fatty acid molecule at a time and transports lipids to specific compartments in the cell [44]. In INS-1 cells, CD36 facilitates FA transport and overexpression induces effects on insulin secretion and FA metabolism, increasing entry and release in β-cells [43].

Recently, expression of a G-protein-coupled receptor (GPCR) specific for medium and long chain saturated FAs, and unsaturated fatty acids, GPR40/FFAR1, was identified, which is expressed almost exclusively in the pancreas (Figure 2) [45,46]. As a GPCR, the binding of FAs to the receptor activates a pathway aiming to activate PKC, and under glucose stimulus in a postprandial state generates oscillations in intracellular Ca^2+^ to stimulate insulin granule release [47]. A study with MIN6 cells showed that FFAR1 regulates the acute potentiation of GSIS induced by palmitate, and the inhibition of FFAR1 during a prolonged exposure with palmitate decreases FA oxidation and positively regulates β-cell function. Therefore, a regulatory effect of the FFAR1 signaling pathway when mediating the deleterious effects triggered by fatty acids is present [48]. Also, studies in GPR40/FFAR1-deficient mice confirmed a role for FFAs in the amplification of insulin secretion; these mice did not develop hyperinsulinemia nor glucose intolerance when given a high-fat diet, since this deficiency protected from the harmful metabolic effects of high-fat feeding [49]. Additionally, downregulation of FFAR1 by RNAi caused impaired FA augmentation of insulin secretion [46]. FFAR1 deficiency protected from hepatic steatosis and hypertriglyceridemia and FFAR1 overexpression led to liver steatosis and, subsequently, impairment of islet function and diabetes [49]. Whether FFAR1 plays a role in β-cell compensation processes and is a possible link between energy surplus and β-cell failure in type 2 diabetes remains to be completely characterized [36].

High levels of FFAs have been proposed as a determinant factor in β-cells apoptosis in different models [50]. Also, recent studies suggest this phenomenon depends on the degree of fatty acid saturation, rather than chain length, in addition to being considered a contributing factor for T2D evolution in patients with obesity [51]. In β-cells, prolonged exposure to high concentrations of long chain FFAs leads to the inhibition of insulin biosynthesis [52] and secretion [53]. Also, palmitic acid inhibits the expression of transcription factor PDX-1 by decreasing DNA binding activity, GLUT-2 transporter [54] and the enzyme acetyl-CoA carboxylase (ACC) expression [31], while increasing the expression of CPT-I [55]. The islet transcription factor PDX-1 was originally discovered as an activator of the insulin and somatostatin genes [56]. Moreover, PDX-1 plays a key role in pancreatic development, regulates transcription of GLUT2 and glucokinase. Mutations in PDX-1 lead to abnormalities in islet function and diabetes in humans and mice [57]. In the PDX-1 heterozygote mouse model and in non-diabetic humans with a mutation in one PDX-1 allele, fasting blood glucose is normal, but there is impairment in insulin secretion and glucose clearance after glucose stimuli [58,59]. Also, PDX-1 heterozygotes mice showed decreased insulin secretion, caused by the inability of the PDX-1 heterozygotes to respond to extracellular glucose, explained by the dramatically reduced expression of glucose transporter GLUT2 [58]. Altogether, these conditions cause an imbalance in the metabolism of the β-cell, generating greater fatty acid accumulation which affects insulin secretion, which is an important factor in the development of diabetes.

## 4. Effects of Saturated Fatty Acids on β-Cells

Palmitic acid (16C) is the highest saturated fatty acid present in the human body. This fatty acid can reduce β-cell proliferation capacity and induce cell death [60]. A critical event in apoptosis development is the release of apoptogenic factors from the mitochondrial intermembrane space to the cytosol, like cytochrome C [61]. Recent studies proposed that proapoptotic proteins such as Bax or Bak interact with the adenine nucleotide translocator (ANT), an internal mitochondrial membrane protein, which facilitates membrane permeabilization and contributes to the release of cytochrome C. It should be noted that palmitoyl-CoA esters are natural ligands of the ANT [62]. For instance, in pancreatic islets of Sprague–Dawley rats under fatty acid stimuli, palmitic acid decreases the expression of ANT, accompanied by cytochrome C release and promoting apoptosis [60].

Another report using pancreatic β-cell culture observed that SFAs including palmitic and stearic acid induce lipoapoptosis, whereas UFAs showed opposite effects. Furthermore, there are differences between cell lines and human islets, for example, human β-cells are more resistant to apoptosis, while in a study with RIN1046-38, both fatty acids induced cellular apoptosis, and in human β-cells, an effect was only seen with stearic acid [51]. Moreover, the accumulation of palmitate-rich triglycerides in the ER of β-cells induced apoptosis, affecting TAG cellular handling and disrupting membrane phospholipid composition [63].

Palmitic acid has been described to generate ER stress, altering the microenvironment of this organelle as a result of the repression of ER-to-Golgi protein trafficking, accumulating unfolded proteins due to protein build-up in the ER lumen [64]. Additionally, the perturbation of membrane lipid composition promotes IRE1 and PERK activation, enhancing dimerization of these transducers since saturated acyl chains are less flexible and interact weakly with transmembrane domains [65]. Also, palmitate modifies the distribution of GRP78/BiP [66], a chaperone responsible for sensing the accumulation of unfolded proteins in the ER lumen [67]. Another study proposed that proinsulin binds to GRP78 and accumulates in the ER of β-cells of mice that had a high fat diet [68]. In INS-1 cells, palmitate treatment induced stored TAGs in the ER, contributing to morphological changes that would promote cell death [63]. The prolonged activation of PERK by palmitate leads to apoptosis via ATF4 overexpression and subsequent CHOP and ATF3 induction. In contrast, all FFAs induce transcription of GRP78/BiP and XBP1 mRNA, which are markers of ATF6 transducer [69].

In a proteomic screen performed to determine changes in the β-cell proteome during ER stress and apoptosis caused by palmitate, analysis showed a link between palmitate and carboxypeptidase-E protein (CPE) levels. CPE is a well-known essential enzyme in the production of insulin, which has been associated with T2D [70,71]. This study reported a protease- and calcium-dependent proteolysis of the CPE protein caused by palmitate treatment [72,73], suggesting a critical effect of palmitate upon insulin processing, mediated by the chronic reduction of CPE levels. Recently, an exome sequencing of morbidly obese women with intellectual disability, T2D and hypogonadotrophic hypogonadism led to the discovery of a new monogenic obesity syndrome with CPE deficiency [74]. An obesity-diabetes syndrome is elicited by a genetic defect in CPE previously described in *fat/fat* and Cpe knockout mouse models [71]. Studies revealed the importance of this enzyme in regulating body weight and metabolism. Likewise, in a study carried out in the INS-1 cell line, β-cells treated with myristic, palmitic and stearic acid, the synthesis of ACC mRNA was inhibited in the basal state and with glucose stimulation; this enzyme is responsible for the formation of malonyl-CoA. However, the mechanism by which the expression of ACC is inhibited is not completely understood. In addition, prolonged exposure to palmitate significantly altered GSIS and suppressed the glucose secretagogue effect. This phenomenon was registered with the exacerbated increase in FA oxidation [53].

In pancreatic islets of Sprague–Dawley rats, it was observed that stimulation with palmitic acid decreases the expression of PDX-1 by 70%, which reduces the expression of GLUT2 and glucokinase, and under prolonged palmitic acid stimulation, insulin mRNA is reduced [54]. Moreover, in isolated rat islets, palmitate did not inhibit PDX-1 expression, but significantly reduced its nuclear localization by sequestration of PDX-1 in the cytosol [75], since its own transcription is regulated by a feedback mechanism [76].

## 5. Effects of Unsaturated Fatty Acids on β-Cells

On the other hand, there is evidence about the protective effect that UFAs exert upon β-cells viability. The response was first defined during the exposure of cells with combinations of saturated and unsaturated FFAs, and improvement observed in the viability reflected a metabolic antagonism between the different fatty acids. This response is probably associated with a condition of molecular competition for the same GPCR (FFAR1) [48]. Under in vitro conditions, the long-chain species were incorporated into TAG molecules, which are composed mostly of SFAs. The progressive accumulation of TAG droplets leads to a physical alteration of cellular architecture within organelles membranes and cell death. Incorporation of UFAs into palmitic-acid-rich TAGs (solid at 37 °C) lowers molecular melting temperatures, increasing fluidity in TAG molecules, and improving TAG cellular metabolism [63]. Also, changes in the composition of phospholipids show implications upon fluidity in membrane systems, such as the ER, the Golgi apparatus and the plasmatic membrane [37], which are relevant to maintaining cellular homeostasis. This could affect membrane signaling, insulin secretion by granule trafficking, fusion of secretory granules to the cell membrane during exocytosis, and protein processing in the ER [63].

For instance, under the treatment of rat insulinoma cells with palmitoleate and palmitate the number of apoptotic cells was lower than those incubated exclusively with palmitate. This suggests that UFAs prevent apoptosis of human β-cells by promoting cell proliferation and maintaining normal expression of ANT [51,60]. The evaluation of different cell lines with different fatty acids showed that SFAs have pro-apoptotic properties, while UFAs maintain protective characteristics. They also concluded that both types of UFAs, MUFAs and PUFAs, are equally effective in preventing apoptosis induced by SFAs regardless of the number of double bonds or chain length, however, MUFAs can be protective at low physiological levels (50 µM) [51]. Under apoptotic conditions, evidence indicates that palmitoleic acid exerts opposite effects compared to palmitic acid and promotes proliferation of β-cells. In addition, it can counteract the toxic effects of palmitic acid. In pancreatic islets of Sprague–Dawley rats, palmitoleic acid did not affect the expression of ANT, and improved parameters of β-cells functionality, increasing GSIS and insulin content in islets. In addition, it prevented the decrease in GSIS and insulin content in islets induced by palmitic acid [60].

Interestingly, n-6 PUFAs have also shown beneficial effects. For instance, ALA supplementation was associated with decreased fasting plasma glucose concentrations in patients [77], higher plasmatic insulin concentrations in nondiabetic participants [78], and a lower prevalence of insulin resistance in normal-weight individuals [79]. However, in overweight or obese patients the protective effects of ALA against insulin resistance were diminished [79]. On the other hand, EPA supplementation in overweight patients with T2D decreased concentrations of fasting plasma glucose, insulin, HbA1c and HOMA-IR [80]. EPA also improved glucose tolerance and decreased plasma glucose [81,82]. Lastly, DHA treatment reduced blood glucose concentrations with enhanced insulin sensitivity in obese diabetic model [83]. Altogether, n-6 PUFA-regulated supplementation could be a therapeutic approach to improve insulin sensitivity in T2D patients.

Evidence shows the possibility that UFAs can promote viability of β-cells under different toxic stimuli. Other authors observed that in cells under MUFAs stimuli, such as palmitoleate, a step in the apoptotic pathway is blocked, i.e., the activation of the effector enzyme caspase-3 [84,85]. Importantly, reports about PUFAs indicate that DHA is associated with anti-inflammatory effects by modulating homeostasis in the ER. In monocytes, DHA treatment has been described to inhibit palmitic acid-induced secretion of proinflammatory interleukins, such as IL-1β [86], however, studies in β-cells are lacking. In other models, DHA inhibits inflammatory pathways and blocks the activation of TLR-4 [21]. Considering that there is a minimal expression of TLR4 in beta cells [87], DHA could possibly show a regulatory role of inflammatory mechanisms as in monocytes, possibly decreasing the inflammatory effect of metabolic overload.

Interestingly, a balance between n-3 and n-6 PUFAs intake has been described. Rats consuming a high n-3/n-6 PUFA ratio diet (1:1, PUFA^1:1^), when compared to SFA-fed rats, had alleviated obesity and lipid stores, as well as decreased serum triglycerides and total cholesterol levels. However, non-significant differences were found between rats consuming the low n-3/n-6 PUFA ratio diet (1:4, PUFA^1:4^) and SFA diets. Also, the PUFA^1:1^ diet enhanced insulin sensitivity decreasing serum fasting glucose and insulin levels, when compared to SFA or PUFA^1:4^ diets [88]. Additionally, in rats fed a PUFA^1:1^ diet, concentrations of TNF-α, IL-6 and C-reactive protein were significantly decreased when compared to SFA-fed rats. Furthermore, in muscle samples from rats fed with the PUFA^1:1^, the expression of TLR-4 protein and mRNA were diminished and non-significant changes in SFA- and PUFA^1:4^-fed rats were found. Collectively, a PUFA^1:1^ diet alleviates insulin resistance and contributes to improvement of obesity in rats by suppressing TLR4 activation [88]. Nevertheless, the effect of TLR-4 activation and insulin processing in β-cells has not yet been studied.

Conversely, in INS-1 cells, a combination of FFAs, including unsaturated fatty acids, caused a minor activation of ER stress signaling. The PERK and IRE1 pathways are activated with oleate and with the combination of palmitate and oleate, but to a lesser extent than palmitate alone [69]. Another investigation with INS-1 cells showed that linoleic, oleic and n-3 fatty acids inhibit the generation of ACC mRNA in its basal state; however, with the stimulation of glucose, it had a greater effect, favoring oxidative catabolism. Also, prolonged exposure to oleic and linoleic acid, as well as to palmitate, markedly altered the insulin response induced by glucose, which authors associated with an increase in FA oxidation [53].

## 6. Final Considerations

A balanced diet is a major contributing factor to health or disease, particularly, T2D which is one of the most relevant pathologies due to its high prevalence and incidence. Several components in diet can modulate T2D development such as dietary fats, specifically, unsaturated fatty acids. The main fatty acid is palmitic acid, which has been known to cause ER stress repressing ER-to-Golgi protein trafficking [64] and, consequently, the accumulation of palmitic-acid-rich TAGs in the ER [63]. Among the most important causes of ER stress is probably the accumulation of new proinsulin associated with GRP78 [68] and proteolysis of CPE [72,73]. Likewise, it reduces GLUT2 expression [54], probably by mechanisms that favor sequestration of PDX-1 in the cytosol, hence, reducing its nuclear localization [75]. Evidence suggests a crucial role of SFAs like palmitic acid in β-cell failure to respond to extracellular glucose causing proinsulin build-up in the ER lumen and generating ER stress. This is a condition which could eventually lead to β-cell apoptosis.

However, UFAs could reverse or prevent the damage generated by excess SFAs. A clear example is palmitoleate, which prevents apoptosis of human β-cells as well as promotes β-cell proliferation and counteracts the deleterious effects of palmitic acid [60]. Indeed, data obtained in our group suggest that palmitoleate treatment inhibits the UPR overactivation (personal communication). In addition, palmitoleate showed anti-apoptotic effects [84,85], improved parameters of β-cell functionality preventing the diminution of GSIS and insulin content in islets induced by palmitic acid [60]. Interestingly, n-6 PUFAs supplementation has been associated with a decrease in fasting plasma glucose, improved glucose tolerance, and, showed an anti-inflammatory activity blocking TLR-4 signaling [21]. Moreover, a high n-3/n-6 PUFA ratio diet (1:1, PUFA^1:1^) treatment has been shown to decrease serum triglycerides and total cholesterol levels and enhance insulin sensitivity and anti-inflammatory effects by reducing inflammatory cytokines [88]. Therefore, a controlled supplementation of PUFAs could become the foundation in the development of optimized treatments and, overall, a deeper knowledge of metabolic pathway alterations which control human body homeostasis; in this case, in hormone-producing cells that regulate energy metabolism.

Several targets could be implied in T2D development and treatment. Such as FFAR1, which plays a key role in β-cell compensatory processes and could be a possible link between energy surplus and β-cell failure in T2D. Targeting PDX-1 and CPE protein [72,73] with UFA treatment could help reduce protein build-up and alleviate ER stress. All this could be achieved by supplementation with UFAs and PUFA^1:1^ to counteract the effects of a high SFA diet, which is a signature condition in T2D and obesity patients. This enhances insulin sensitivity, diminishes inflammation and, overall, improves β-cell function. Although further information is required to confirm the therapeutic role of UFAs in disease.

## 7. Conclusions

SFAs have been broadly related with cell apoptosis and this has been associated with several metabolic diseases including T2D. Specifically a decrease in β-cell mass caused by an increase in apoptosis has been linked to palmitic acid. Lipotoxicity triggered by palmitic acid affected the response of β-cells to extracellular glucose causing proinsulin build-up and generating ER stress and therefore origin an imbalance in energetic metabolism of the body. On the other hand, UFAs such as palmitoleic and oleic could reverse or prevent the damage generated by excess of SFAs. MUFAs have shown to control β-cell parameters such as GSIS and insulin content. Also, a controlled supplementation of PUFAs has shown to optimized plasmatic cholesterol and triglycerides levels and enhance insulin sensitivity. This could lead to the development of healthier and improved dietary treatments of patients with T2D, taking into account de novo lipogenesis as an important source of palmitoleate is altered. Further information is required to confirm the therapeutic role of UFAs in disease. Nonetheless, evidence suggests unmistakably that a dietary approach, instead of pharmacological, could improve and possibly prevent the development of T2D and other metabolic diseases. This review raises the importance of a balanced fatty acids diet in health and disease, suggesting alternative therapeutic approaches against diabetes.
